# Effect of Multileaf Collimator Leaf Position Error Determined by Picket Fence Test on Gamma Index Value in Patient-Specific Quality Assurance of Volumetric-Modulated Arc Therapy Plans

**DOI:** 10.7759/cureus.12684

**Published:** 2021-01-13

**Authors:** Cemile Ceylan, Serpil Yondem Inal, Elif Senol, Berrin Yilmaz, Sevim Sahin

**Affiliations:** 1 Radiation Oncology Department, Istanbul Oncology Hospital, Istanbul, TUR; 2 Health Sciences Institute, Yeditepe University, Istanbul, TUR; 3 Radiation Oncology Department, Memorial Bahcelievler Hospital, Istanbul, TUR; 4 Medical Imaging Department, Fenerbahçe University, Istanbul, TUR

**Keywords:** mlc error, patient-specific quality assurance, gamma-index evaluation

## Abstract

Aim

The correlation between the MLC QA (IBA Dosimetry, Germany) results of the picket fence test created with intentional errors and the patient's quality assurance (QA) evaluation was investigated to assess the impact of multileaf collimator (MLC) positioning error on patient QA.

Materials and methods

The picket fence, including error-free and intentional MLC errors, defined in Bank In, Bank Out, and Bank Both were analyzed using MLC QA. The QA of 15 plans consisting of stereotactic radiosurgery (SRS), stereotactic body radiotherapy (SBRT), and conventionally fractionated volumetric-modulated arc therapy (VMAT) acquired with electronic portal imaging devices (EPID) was evaluated in the presence of error-free and MLC errors. The QA of plans were analyzed with 2%/2 mm and 3%/3 mm criteria.

Results

The passing rates of the picket fence test were 97%, 92%, 91%, and 87% for error-free and intentional errors. The criterion of 3%/3 mm wasn’t able to detect an MLC error for either SRS/SBRT or conventionally fractionated VMAT. The criterion of 2%/2mm was more sensitive to detect MLC error for the conventionally fractionated VMAT than SRS/SBRT. While only two of SBRT plans had <90%, four of conventionally fractionated VMAT plans had a <90% passing rate.

Conclusion

We found that the systematic MLC positioning errors defined with picket fence have a smaller but measurable impact on SRS/SBRT than the VMAT plan for a conventionally fractionated and relatively complex plan such as head and neck and endometrium cases.

## Introduction

Intensity-modulated radiation therapy (IMRT) and volumetric-modulated arc therapy (VMAT) have become a standard modality for delivering highly conformal dose distributions as compared to 3D conformal techniques. Both delivery modalities are capable to obtain highly conformal dose distribution into the target and steep dose gradients within the irradiated volume while minimizing doses in the organ at risk (OAR). VMAT is a relatively new dose delivery technique with fewer monitor units and shorter treatment time as compared to the IMRT delivery technique [1]. The highly modulated dose distribution in VMAT plans can be achieved with the accurate operation of gantry rotation speed, the accuracy of multileaf collimator (MLC) leaves movement, and dose rate variation during radiation delivery [2]. Because of the high complexity and accuracy in the dose delivered with the MLC shape, gantry speed, and dose rate changing during one or more gantry rotations of the IMRT and VMAT treatment plans, patient-specific pretreatment quality assurance (QA) is generally considered a vital and prerequisite to patient treatment. The lack of MLC position accuracy, even in case of small positional deviations between planned and delivered MLC shapes during radiotherapy can lead to significantly inaccurate dose distributions in the patient’s treatment plan affecting the intended doses of the tumor and OARs [3-5]. Rangel et al. reported that a 1 mm error in the position of MLC leaves could cause dose differences in the equivalent uniform dose (EUD) up to 5.6% for clinic target volume (CTV) of head and neck patients’ plans that have high modulation [5]. Another study done by Oliver et al. analyzed the MLC positioning error effect for VMAT plans and they concluded that the MLC positional errors in VMAT treatments should be within ±0.6 mm to keep the dose accuracy within ±2% [6]. Therefore, the quality assurance (QA) programs of MLC performance to determine the accuracy and errors in leaf position are essential for the accurate delivery of IMRT/VMAT plans for each fraction.

Several authors have recommended that the accuracy of MLCs positioning should be within 2 mm to achieve negligible dosimetric effect in sliding window treatments. Therefore, the beam interlock tolerance level in terms of MLC positioning error of most linear accelerators has been adapted according to this recommendation [7-9]. In clinical applications, if a leaf is detected to be outside the tolerance, the beam is held off until leaves move to the correct position, and then the machine continues to beam on again. Therefore, in case of the MLCs positioning errors that will not trigger MLC interlock during beam delivery, dosimetric errors that might occur in IMRT/VMAT and especially SBRT/SRS plans having small and highly modulated segmented MLC-shaped fields were unknown. Although the machine interlock value for MLC positioning generally is set to 2 mm, Budgell et al. reported that the accuracy of leaf positioning should be better than 1 mm for accurate IMRT delivery [10]. The American Association of Physicists in Medicine (AAPM) and the European Society of Radiotherapy and Oncology (ESTRO) recommend within their reports that ±1 mm and ±0.5 mm as the acceptance criteria for MLC leaves positions, respectively [11-12].

Different methods have been prescribed to examine MLC quality assurance in the literature. One of them is to analyze the machine’s logfile to perform MLC quality control, including leaf position, gantry angle, leaf speed, and even dose reconstruction of the patient [13-14]. Another MLC quality assurance method is the conventional method for checking leaf position accuracy with different images of a dynamic MLC pattern. In this method, either two dimensional (2D) images obtained from radiographic/Gafchromic film or digital images provided by electronic portal imaging devices (EPID), 2D diode, arrays were analyzed to define MLC position error by either visual or digital analysis using different software [15-18]. When both methods are compared using the scanned film by visual inspection is a subjective and relatively inaccurate and time-consuming method than analyzing a digital image, which is obtained quickly from EPID. Therefore, the clinical usage of analyzing EPID images was accepted more efficiently and more easy to repeat than film.

The picket fence pattern, which is based on the definition of leaf position by leaf pairs moving through the designed pattern with sliding slit as stopping at a number of equally spaced positions creating narrow irradiated area is well known and widely used. The picket fence pattern is the most common test to measure the position error of each MLCs and compare it with other leaves alignments for either film or EPID image inspection. There are two methods to conduct the picket fence, which are either using a uniform pattern with abutting fields or creating narrow bands with the interval. Rowshanfarzad et al. suggested that the MLC performance test with the picket fence test for IMRT/VMAT based on their EPID based investigation should be done daily, after the machine warm-up or before patient-specific QA to define the discrepancy between treatment plan and delivery dose distributions [17]. 

In this study, we purposed to evaluate the dosimetric error within VMAT plans for SRS/SBRT applications caused by MLC leaf position errors determined by picket fence created by narrow bands that will not trigger the linear accelerator MLC interlock. The detection of an MLC positioning error with a daily picket fence test, which is a rigorous and comprehensive machine-specific quality assurance procedure, could help reduce the need for patient-specific quality control for IMRT/VMAT delivery.

## Materials and methods

Delivery system and designs of MLC positions for picket fence tests

All measurements, including the picket fence test and quality assurance of patient plans, were performed using the Versa HD Signature (Elekta Oncology Systems, UK) linear accelerator equipped with an Agility MLC system (Elekta AB, Stockholm, Sweden). The Agility MLC consists of 160 MLCs placed on two leaf banks of 80 leaves each with leaf width 5 mm and its capabilities to create an optimized delivery with 1 mm increments of each leaf’s placement. Furthermore, the 5 mm leaf width can be reduced in the jaw direction by positioning the Y-jaws within leaf widths on either end of the target in steps of 1 mm [19]. The Agility MLC has the capability of interdigitation. The position of each leaf determines using an optical system via the attached reflector at each leaf end and illuminated with a light source. The leaf positions were adjusted by tuning the leaf offset values in case of leaf position error occurred.

The megavoltage image acquisition at Versa HD Signature system was done by the iView GT EPID (Elekta AB) when the detector dimension is 41x41 cm^2^. The iView GT system has a source-detector distance (SDD) of 160 cm and the maximum field size at the isocenter is 26x26 cm^2^ sensitive area containing 1024x1024 pixels. Six MV and 6 FFF photon energies were used for treatment plans and picket fence test irradiation. Before the irradiation of picket fence fields and treatment plans, calibration of MLCs and iView GT, including alignment, correlation of radiation, and EPID isocenter, as an essential requirement for SRS/SBRT delivery, SDD agreement tests were done by the service engineer.

**Figure 1:**
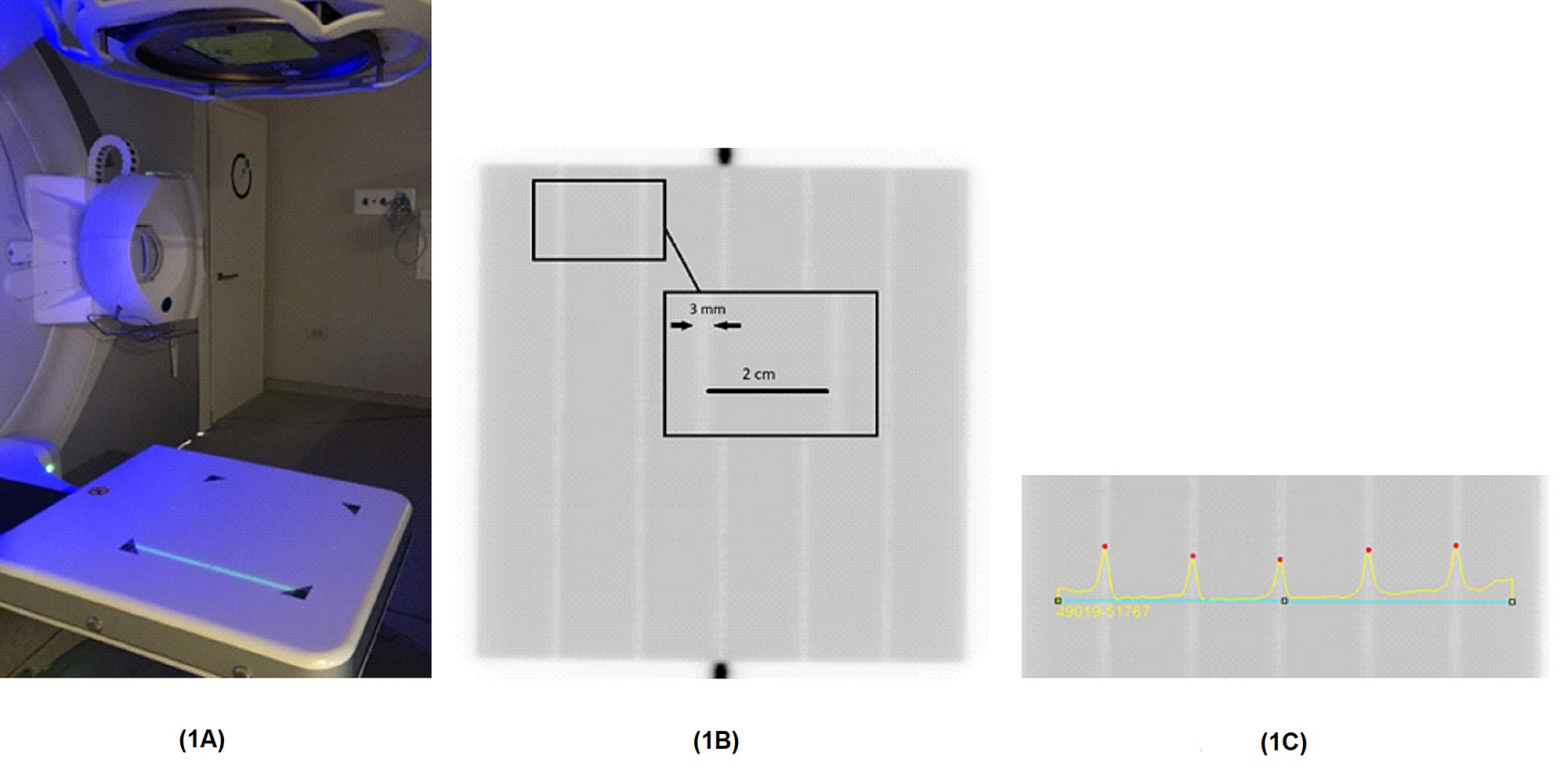
(1A) The measurement of the picket fence test at G=0 with iViewGT. (1B) EPID images of the picket fence test without an intentional MLC error, which is accepted as a reference test to compare the other picket fence tests with intentional leaf errors. (1C) The analysis of the reference image obtained at G=0 represents the summation of the image intensity values across the right-left direction of the machine. The peaks of the profiles show the center of the fences. EPID: electronic portal imaging device; MLC: multileaf collimator

The picket fence leaf pattern introduced by Chui et al. was used for the definition of leaf position accuracy and the reproducibility of the individual leaf position [20]. The picket fence test allows the evaluation of the positioning error of each leaf in terms of the measurement of the distance between mutual leaf pairs either by visual inspection or by measuring the distance between peaks on the profiles using the specific software. The measurement setup using the EPID system of the linear accelerator, which was positioned at 160 cm SDD, and the acquired reference picket fence image at the gantry 0^o^ position is as shown in Figure 1A. In this study, the leaves moved to a stop at every 2 cm and formed six pickets of size 0.3 cm × 20 cm (Figure 1B). The beam was delivered at gantry angles of 0^o^ and 90^o^ as reference measurements of the picket fence without any intentional leaf error. Figure 1C represents the peaks of the profile taken across the right-left direction of the linear accelerator (LINAC) corresponding to the maximum intensity value into each fence of the test. Furthermore, the reference picket fence images were obtained immediately after MLC calibration. The picket fence test image acquired from EPID was loaded to MLC QA (IBA Dosimetry, Germany) software, which is a commercially available software to analyze which selected pickets required criteria recommended by the AAPM Task Group 142 [11].

Following the deliveries of the reference picket fence tests, picket fence tests with three different intentional leaf errors have been performed to compare with the result of the reference picket fence test in the MLC QA software. Three types of intentional errors were introduced in the picket fence test named Bank in an opening offset of 1 mm on the leaf bank Y1 toward the isocenter, then a closing offset of 1 mm on the leaf bank Y2 toward the outside of the isocenter named Bank Out, and an opening offset of 1 mm for both leaf banks, Both Out, on all six fences of the picket fence test. The consequences of the intentional errors were either an enlargement or a narrowing of the picket fence gap relative to an error-free picket fence. Each picket fence irradiation was carried out five times to reduce the uncertainties and calculate the standard deviation of positioning errors using the MLC QA plug-in software.

MLCQA software for the determination of MLC positioning errors

Generally, the evaluation of the picket fence images acquired by EPID or film can be done using homemade software or commercial programs to verify if MLC positioning accuracy is within the Task Group TG-142 recommendations [11]. MLC QA is one of the commercial tools, and it supplies routine MLC quality assurance tests to define MLC positing error and speed error according to the TG-142 report [11]. The full-width half-maximum (FWHM) value between MLC strips and the distances between gaps within the picket fence test images were measured and compared with criteria according to TG-142 to evaluate the positioning error of the leaves. The result of each picket fence image evaluation is presented as a percentage of the deviation of the peak position of each leaf pair from the average of all peak positions in the picket to define error for each leaf pair and the distance (gap width) between opposing leaves for each leaf pair. The result of each picket fence test with an intentional MLC position error was compared with error-free imaging of the reference picket fence test.

Description of SRS/SBRT and VMAT plans and patient-specific QA with iViewDose

In this study, the consequences of the intentional MLC positional errors defined by the picket fence test analysis were investigated for association with the result of patient-specific quality measurements, which were delivered with the same intentional MLC errors for the picket fence test. A total of 15 VMAT plans, including five SRS planning of brain metastasis, five SBRT planning of vertebra, lungs, and liver cases, and five VMAT planning of lungs, prostate, head and neck, and brain tumor cases with conventional fraction were included for investigation of the impact of MLC position errors. All plans were generated in the Monaco v5.11.02 treatment planning system (Elekta Corporation, Atlanta, GA) using the Monte Carlo dose calculation algorithm. SRS/SBRT plans were designed with 6 MV FFF photon energy with a calculation grid size of 1 or 2 mm. VMAT plans for conventionally fractionated treatment were designed with 6MV photon energy with a calculation grid of size 3 mm.

The comparison of EPID reconstructed with the planned dose distribution was done by a 3D ɣ-evaluation method (3% global dose difference and 3 mm distance-to-agreement) using the mean ɣ value, the maximum 1% ɣ value, and the percentage of points with ɣ < 1 within the 50% isodose surface of the planned maximum dose. In addition, the difference between the measured and planned total fraction dose at the dose reference point (∆DRP %), which was chosen as the isocenter of the plans, was evaluated. The passing rate with these criteria of each patient plan with an intentional error was compared with the result of plans without an intentional MLC error related to the magnitude of the impact of MLC positioning error. The tolerance level of the passing rate was set to 95%, which is used in the clinical acceptance of IMRT/VMAT plans' quality assurance. The correlation of MLC misalignment impact on the dose distribution between different MLC misalignments was performed by a comparison of the passing rate of each iViewDose result, which was obtained with and without MLC positioning errors. Table 1 shows the summary of the evaluated patients’ plans, including types of treatment approaches, the minimum segment size that the small segment size leans to have a variation in the MU calculation in the presence of even a minimum MLC positioning error.

**Table 1:** The summary of the selected and evaluated VMAT plans for SRS/SBRT and conventionally fractioned radiation therapy treatments SRS: stereotactic radiosurgery; SBRT: stereotactic body radiotherapy; GBM: glioblastoma multiforme; VMAT: volumetric-modulated arc therapy

Treatment Modality	Treatment Region	Calculation Voxel Size (mm)	Statistical Uncertainty	Arc Number	Segment Number	Minimum Segment Width (cm)
SRS_1	Cranial	1	1% per calculation	1	173	0.5
SRS_2	Cranial	1	1% per calculation	1	139	0.5
SRS_3	Cranial	1	1% per calculation	2	95	1
SRS_4	Cranial	1	1% per calculation	2	58	1
SRS_5	Cranial	1	1% per calculation	3	154	1
SBRT_1	Lumbar vertebrae	2	1% per calculation	3	422	0.5
SBRT_2	Lung	2	1% per calculation	3	215	0.5
SBRT_3	Liver	2	1% per calculation	4	145	0.5
SBRT_4	Lung	2	1% per calculation	2	143	0.5
SBRT_5	Lumbar vertebrae	2	1% per calculation	2	76	1
fVMAT_1	Lung	3	1% per calculation	1	110	1
fVMAT_2	Prostate	3	1% per calculation	1	208	0.5
fVMAT_3	GBM	3	1% per calculation	3	179	0.5
fVMAT_4	Larynx	3	1% per calculation	2	266	0.5
fVMAT_5	Endometrium	3	1% per calculation	2	280	0.5

## Results

The results of the picket fence tests using MLC QA software

**Figure 2:**
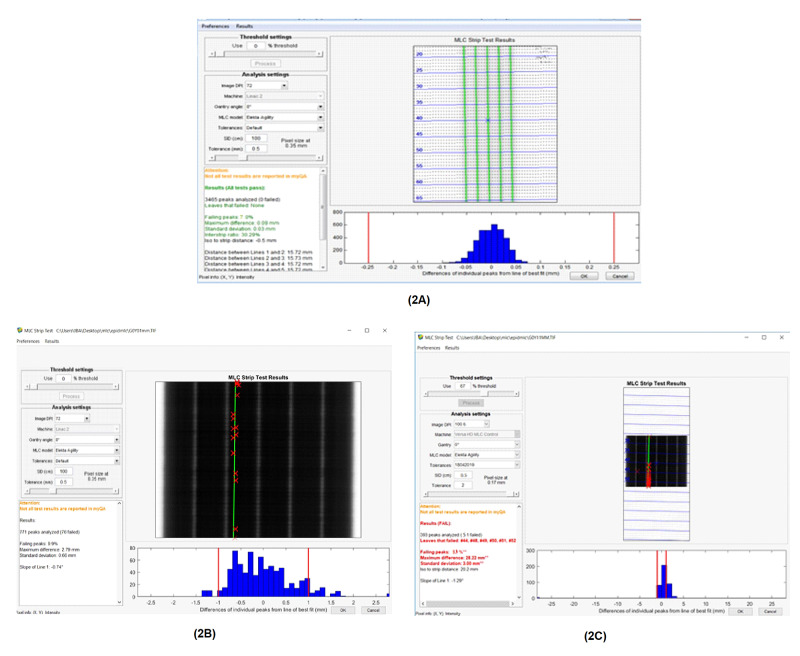
A screen capture of MLC QA software showing the analysis results of the picket fence tests. (2A) The result of error-free picket fence test-taking as a reference test with having 97% passing rate. (2B) and (2C) represent the results of the Bank In and Both Out picket fence tests that having the passing rates were 91% and 87%, respectively.

The evaluation of the MLC error was investigated using MLC QA software, which has an algorithm to detect the position of each leaf from picket fence images searching with the FWHM value between each peak as presented in Figure 1C. The detectable disposition of each leaf at the picket fence images, including the intentional MLC errors, named Bank In, Bank Out, and Both Out, was calculated by comparing error-free picket fence test image. A series of graphical results obtained by MLC QA software is shown in Figure 2 for the types of intentional MLC positioning errors introduced into the picket fence test and the evaluation of its impact was presented with the passing rate of the resultants according to TG-142 criteria [11].

The leaf position results on the picket fence images of the reference test without error, the test image of Bank In error, the test image of Bank Out, and the last test image of Both Out were 97%, 91%, 91%, and 87% of passing rate, respectively. These results were verified with the five times of each measurement to eliminate the effect of the random error of MLCs.

Result of MLC misalignment magnitude effect on the passing rate of a gamma-index method of selected treatment plans

MLC quality assurance based on the picket fence test, which has intentional errors to define the impact of an MLC positional error on dose distribution was conducted for patient-specific QA of 15 cases treated with the VMAT delivery technique. The EPID based dose verification software iViewDose was used to measure the VMAT plan dose distributions for different approaches to treat patients such as SRS and SBRT with MLC error-free and intentionally introduced MLC errors.

**Figure 3:**
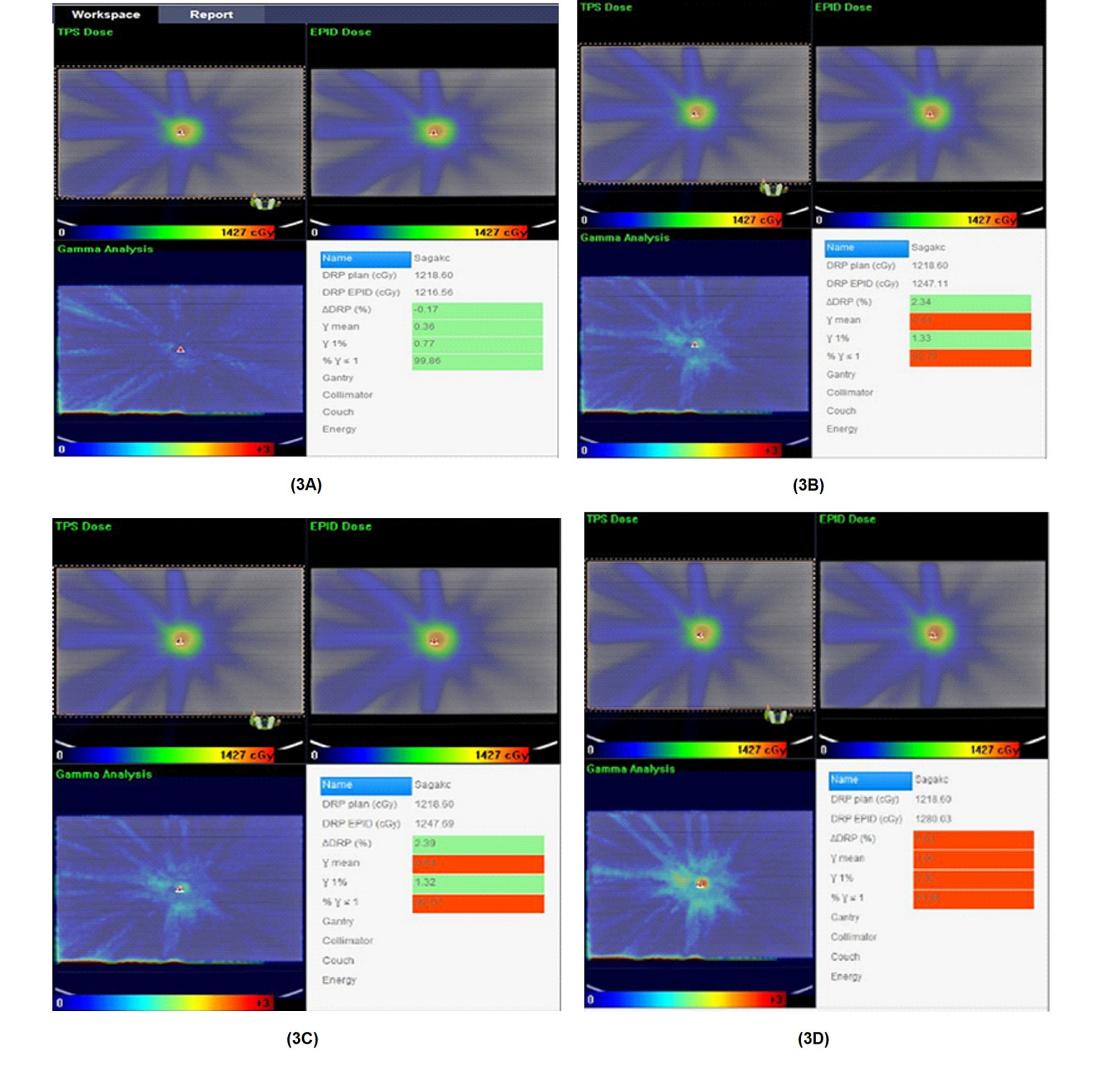
(3A), (3B), (3C), and (3D) represent the dose distribution for SBRT lung case calculated by the iViewDose dosimetry system, which was evaluated with a gamma index of 2% dose and 2 mm distance agreement criterion for error-free, Bank In error, Bank Out error, and Both Out error, respectively SBRT: stereotactic body radiotherapy

In this study, the gamma-index method criterion for SRS/SBRT and conventional fractionated VMAT plans could achieve greater than 95% passing rates in MLC error-free plans except for a plan for an endometrium case, which has a 93% passing rate for the conventional criterion of 3%/3mm. The results of patient-specific quality assurance with iViewDose can be seen in Table 2. The resultant dose distributions with MLC positioning errors were compared with the original plan dose distribution with an error-free reference dose distribution. Figure 3 presents the evaluated dose distributions of a VMAT plan belonging to the patient who was treated with SBRT according to the 2%/2 mm of gamma criteria having the passing rate (γ≤1) values of 99.86%, 92.79%, 92.57%, and 53.96% in the presence of error-free, Bank In error, Bank Out error and Both Out errors, respectively. The passing rates with 3%/3 mm of gamma criteria results of the same patient’s SBRT plan dose distribution were 100%, 99.9%, 99.9%, and 90.9% for measurements with error-free, Bank In error, Bank Out error, and Both Out errors, respectively.

**Table 2:** The percentage of passing rate for all cases calculated with two different gamma index evaluation criteria concerning the magnitudes of MLC misalignments of error-free, Bank In, Bank Out, and Both Out MLC: multileaf collimator

			DD(%):3, DTA(mm):3				DD(%):2, DTA(mm):2			
Patient	Treatment Site	Evaluation Criteria	Error Free	Bank In	Bank Out	Both Out	Error Free	Bank In	Bank Out	Both Out
SRS1	Brain	% γ ≤ 1	96.9	97.7	97.4	98	92.4	93.7	93.8	84.1
		∆DRP %	0.2	1.4	1.3	3	0.2	1.4	1.3	3
SRS2	Brain	% γ ≤ 1	99.8	99.7	99.7	93.9	98.8	96.4	96.5	78
		∆DRP %	-0.5	2.4	2.3	5.3	-0.5	2.4	2.3	5.3
SRS3	Brain	% γ ≤ 1	100	99.9	99.7	99.5	99.4	98.4	98.3	93.4
		∆DRP	2.2	2.5	2.6	3.5	2.2	2.5	2.6	3.5
SRS4	Brain	% γ ≤ 1	99.9	100	100	98.2	98.9	97.4	97.8	78.3
		∆DRP	-1.4	0.2	0.1	2.3	-1.4	0.2	0.1	2.3
SRS5	Brain	% γ ≤ 1	99.8	100	100	100	98.9	99.7	99.5	98.8
		∆DRP	-0.5	0.6	0.4	1.3	-0.5	0.6	0.4	1.3
SBRT1	Lumbar vertebrae	% γ ≤ 1	99.8	92	82.2	67.7	94.5	68.3	68.8	26
		∆DRP %	1	5.9	7.2	13.9	1	7.7	7.2	13.9
SBRT2	Lung	% γ ≤ 1	99.8	88.5	87.9	60.3	95.9	69.3	69.5	22.7
		∆DRP %	2.4	8.8	9	15.7	2.4	8.8	9	15.7
SBRT3	Liver	% γ ≤ 1	99.4	98.8	98.8	89.8	96.4	91.4	91.8	64.7
		∆DRP %	1.3	3.9	3.9	6.2	1.3	3.9	3.9	6.2
SBRT4	Lung	% γ ≤ 1	100	99.9	99.9	90.9	99.9	92.8	92.6	54
		∆DRP %	-0.2	2.3	2.4	5	-0.2	2.3	2.4	5
SBRT5	Lumbar vertebrae	% γ ≤ 1	99.4	99.1	99	94.4	94.1	96.9	95.9	79
		∆DRP %	-2.8	-1.6	-1.5	0.4	-2.8	-1.6	-1.5	0.4
VMAT1	Lung	% γ ≤ 1	99	99.4	99.3	96	94.8	94.4	95.3	81.5
		∆DRP	-0.5	1.4	1.2	2.8	-0.5	1.4	1.2	2.8
VMAT2	Prostate	% γ ≤ 1	99.9	83.6	83.1	63.8	97.1	69.6	69.3	27.5
		∆DRP	1.6	5	5.1	8.3	1.6	5	5.1	8.3
VMAT3	Brain	% γ ≤ 1	96.6	92.1	94.1	74	77.9	78.2	81.5	50.8
		∆DRP	-1.1	3.7	3.3	5.9	-1.1	3.7	3.3	5.9
VMAT4	Head and Neck	% γ ≤ 1	98.2	62.4	63.3	25.8	91.1	34.7	36.3	7.8
		∆DRP	-0.4	10	9.6	17.6	-0.4	10	9.6	17.6
VMAT5	Endometrium	% γ ≤ 1	93.3	55.5	55.2	25.8	79.2	34.1	31.5	6.5
		∆DRP	1	4.5	5	8.9	1	4.5	5	8.9

**Figure 4:**
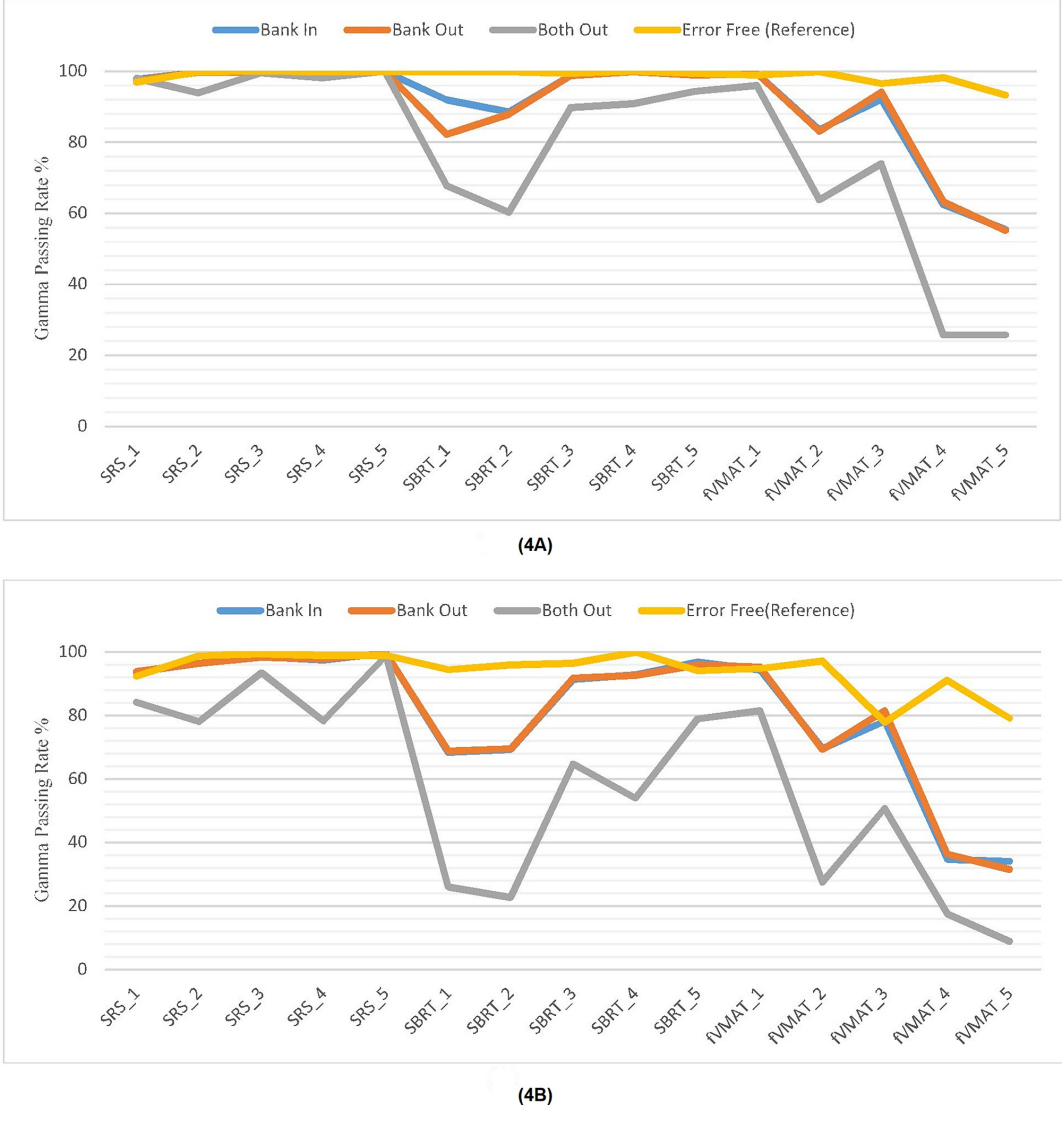
Decreases of the gamma passing rates with different gamma evaluation criteria. which are (4A) 3%/3 mm and (4B) 2%/2 mm according to the magnitudes of MLC positioning errors MLC: multileaf collimator

Although the criterion of 3%/3 mm has generally been used for gamma evaluation for conventionally fractionated VMAT/IMRT QA, the stricter gamma criterion of 2%/2 mm has also been selected for evaluation dose distribution for both conventionally fractioned and SRS/SBRT patient-specific VMAT QA. As shown in Figure 4, the gamma passing rates resulted in error-free, Bank In, Bank Out, and Both Out situations of MLCs and were evaluated with gamma criterion of 2%/2 mm and 3%/3 mm for all included cases. It can be recognized that the maximum effect was presented as the same magnitude of intended MLC position errors effect on the VMAT plans for conventionally fractionated endometrium and head and neck cases. If the gamma criteria were even set to 3%/3 mm, the skewness of the gamma passing rates of the endometrium case was 98.2%, 62.4%, 63.3%, and 25.8% in case of error-free, Bank In, Bank Out, and Both Out, respectively. The smallest segment area at the plan of this endometrium patient consisting of high modulation was 0.5 cm.

The changing of the passing rate of the gamma index belonging to all plans with various gamma criteria related to the magnitudes of MLC misalignments of Bank In, Bank Out, and Both Out is illustrated in Figure 4. Furthermore, Table 2 summarizes the changing of the gamma index due to MLC positioning errors for all cases. As shown in Table 2, if the gamma criteria of 3%/3 mm were selected to evaluate SBRT/SRS plans, the effect of leaf positioning errors, which were set to a maximum 2 mm offset, could not be detected. Thus, the gamma evaluation had a little deviation from the 95% passing rate.

As a result of the evaluation using gamma criteria of 3%/3 mm, all plans of SRS/SBRT with intentional MLC positioning errors were accepted as clinically applicable except three cases, which were the lung, lumbar vertebrae, and liver having 67.7%, 60.3%, and 89.8% passing rates in the presence of the Both Out error.

Correlation of the picket fence test passing rates with the gamma evaluation passing rate of selected treatment plans resulted in occurring MLC positioning errors

The correlation between MLC QA results, with or without intentional errors and the gamma passing rate of patient-specific QA in the clinical VMAT plans, was quantitatively analyzed to determine how the patient-specific QA will be affected by the MLC positioning error. Table 3 summarizes the passing rates that the measured dose distributions were compared with and without leaf position errors and pass rates calculated under a criterion of 2%/2 mm or 3%/3 mm for each case.

**Table 3:** The passing rates of VMAT plans using two types of gamma criteria depend on the magnitude of MLC misalignments VMAT: volumetric-modulated arc therapy; MLC: multileaf collimator

	% γ ≤ 1, The passing rate for Picket Fence passing rate 97% (Error Free)		% γ ≤ 1, The passing rate for Picket Fence passing rate 92% (Bank In)		% γ ≤ 1, The passing rate for Picket Fence passing rate 91% (Bank Out)		% γ ≤ 1, The passing rate for Picket Fence passing rate 87% (Both Out)	
	3%,3 mm	2%,2 mm	3%,3 mm	2%,2 mm	3%,3 mm	2%,2 mm	3%,3 mm	2%,2 mm
SRS1	96.9	92.4	97.7	93.7	97.4	93.8	98	84.1
SRS2	99.8	98.8	99.7	96.4	99.7	96.5	93.9	78
SRS3	100	99.4	99.9	98.4	99.7	98.3	99.5	93.4
SRS4	99.9	98.9	100	97.4	100	97.8	98.2	78.3
SRS5	99.8	98.9	100	99.7	100	99.5	100	98.8
SBRT1	99.8	94.5	92	68.3	82.2	68.8	67.7	26
SBRT2	99.8	95.9	88.5	69.3	87.9	69.5	60.3	22.7
SBRT3	99.4	96.4	98.8	91.4	98.8	91.8	89.8	64.7
SBRT4	100	99.9	99.9	92.8	99.9	92.6	90.9	54
SBRT5	99.4	94.1	99	96.9	99	95.9	94.4	79
VMAT1	99	94.8	99.3	94.4	99.3	95.3	96	81.5
VMAT2	99.9	97.1	83.1	69.6	83.1	69.3	63.8	27.5
VMAT3	96.6	77.9	94.1	78.2	94.1	81.5	74	50.8
VMAT4	98.2	91.1	63.3	34.7	63.3	36.3	25.8	7.8
VMAT5	93.3	79.2	55.2	34.1	55.2	31.5	25.8	6.5

Figure 4 shows the dependency of the gamma passing rates of patient-specific QA with different gamma evaluation criteria in case of intentional MLC positioning errors. The evaluation of the gamma values of all cases resulted in exceeding 95% by the gamma criteria of 2%/2 mm except for four conventionally fractionated VMAT cases that have high modulation. Furthermore, when the picket fence test results of the Bank In and Bank Out errors were obtained slightly under the accepted passing rate value of 95% such as 92% and 91% and MLC positioning errors were considered tolerable since the machine state was not triggered from Beam On to Beam Off. Nine out of 15 cases, including one of SRS plans, four of SBRT plans, and four of conventionally fractionated VMAT plans had clinically unacceptable passing rates of the gamma evaluation by the gamma criteria of 2%/2 mm. The MLC QA result of the Both Out MLC positioning error situation was 87%, which was under the accepted threshold. Consequently, the passing rates of all cases were found unacceptable based on 2%/2 mm gamma evaluation criteria except one SRS case treated for recurrent high-grade brain tumor having an approximately 99% passing rate for all MLC error scenarios. These results, as shown in Figure 4, indicate that the VMAT plans for conventionally fractionated patients having higher modulation than VMAT plans for SRS/SBRT patients are the most sensitive to MLC leaf positioning errors.

## Discussion

In this study, we investigated an EPID-based method using MLC QA software to detect abutment leaves in terms of positional errors of MLC that would adversely impact the patient-specific QA of VMAT plans. The evaluation of the picket fence tests for MLC positioning error-free, Bank In, Bank Out, and Both Out cases was done to detect MLC positioning errors by MLC QA software according to TG-142 suggestions. The impact of an MLC positioning error on IMRT/VMAT plans was studied by several authors by recalculating dose distribution in the treatment planning system (TPS) to reveal dose differences on target and critical structures [21-22]. Unlike those studies to eliminate errors sourced by the TPS such as beam modeling uncertainties, we tried to directly correlate the effect of MLC positioning error with the gamma passing rate of patient-specific QA for VMAT plans considering clinically acceptable gamma criterion. Although MLC QA software can detect MLC positioning errors less than 1 mm by calculating the FWHM value between each leaf, we generated an intentional error of 1 mm in the positioning error of MLC for each picket fence as systematic leaf position errors that are not able to trigger the linear accelerator to become beam-off status during the delivery of VMAT. The passing rate values were subtracted as a result of picket fence tests by calculating the difference between the predetermined positions of the MLCs in terms of fence intervals of the picket fence tests and the calculated position using the acquired EPID image of each test containing the intentional errors. Although the picket fence test was routine for the monthly or weekly checking procedure to define MLC misalignment error, a daily QA procedure of the quantitative analysis of the picket fence test using EPID images in combination with MLC QA software could allow us to predict MLC positing errors that would cause changes in the QA of the patient plan. Thus, a very limited study had been performed to investigate patient-specific QA results for SRS/SBRT with related MLC positioning errors correlated with the resultant of the picket fence test. We recently focused on the investigation of the effect of intentionally applying MLC positioning errors on patient plans, including SRS/SBRT and conventionally fractionated VMAT deliveries. The correlation between the MLC QA result that is subtracted from quantitative analysis of the picket fence tests’ image acquired by EPID in presence with or without intentional errors and the gamma passing rate of the patient-specific QA of VMAT plans were evaluated to determine how the patient-specific QA will be affected by the MLC positioning error.

Currently, accurate MLC testing is time‐consuming, which limits the feasibility of higher frequency testing in a clinical setting. Streamlining quantitative MLC QC and the analysis of the result using automated tools enable performance assessment and are the first steps toward MLC performance optimization. The MLC QA is an essential procedure to routinely control MLC leaf positioning for complete control of the patient plan containing complex treatment fields since any error in leaf positioning can cause significant dose delivery errors. Therefore, IMRT and VMAT require extensive knowledge of the MLC's position accuracy and repeatability. To achieve better than 1 mm accuracy and precision of MLC leaf positioning, routine MLC QA testing is recommended by TG-142 [11] to be performed weekly using visual inspection of matched segments and monthly quantitative testing using a special test pattern such as a picket fence test described by Losasso [3]. Different methods have been described in the literature for MLC QA to deliver IMRT/VMAT plans accurately [18,23-26]. One of the used methods for MLCQA for IMRT/VMAT delivery was the two-dimensional (2D) evaluation of either film or scanned image by visual inspection, which are subjective, time-consuming, sensitive to external conditions, and a less accurate way to define MLC positioning errors [25]. Another method that does not require physical measurements for MLCQA, gantry angle, collimator angle, and stored cumulative dose per control point would be retrospectively analyzed via the MLC log files recorded by the machine end of each IMRT/VMAT delivery. Agnew et al. compared the MLC positioning errors' detection capability of machine log files and the EPID images of the picket fence tests for IMRT and VMAT deliveries that the trajectory logs created during the delivery of a picket fence test did not detect leaf positional errors that were detected using an in-house EPID based software [27]. The different 2D array detectors, such as MapCheck (Sun Nuclear, Melbourne, FL), 2D-ARRAY seven29 (PTW, Freiburg, Germany), and MatriXX (IBA Dosimetry, Germany) have also been used to define MLC positioning error by the picket fence test [26,28-29]. The major concerns about these devices are their low spatial resolution and insufficient sensitivity to detect an MLC positioning error when errors are smaller than 2 mm and 1 mm in the criterion of the 3%/3 mm and 2%/2 mm gamma index, respectively. Previous studies demonstrated that relatively small random MLC positioning errors, such as those smaller than 0.3 mm had insignificant dosimetric changes in targets and OARs, while MLC positioning errors that were created intentionally to obtain systematic errors even smaller than 1 mm can cause a significant impact on the dose distribution depending on the complexity of the IMRT/VMAT plan. Although TG-142 [11] and ESTRO 2008 Reports [12] recommend defining MLC positioning accuracy with qualitative weekly and quantitative monthly QA, an independent quality assurance program with high accuracy for MLC QA to be performed daily, as suggested in a recent study, can adequately detect clinically signiﬁcant MLC positioning errors to provide the accurate delivery of IMRT and VMAT. Therefore, the digital image of the picket fence test acquired by EPID can be used for analysis to quantitatively assess MLC positioning errors by either commercial or in-house software, which is a widely accepted sensitive method as the film [17].

A method has been presented in this study for the detection of MLC positioning errors with the picket fence test, which provides early warning for the effect on patient-specific QA that cannot be detected according to the gamma index value used in clinical evaluation. As mentioned before, an advantage of the proposed method is that the users can verify the effect of MLC leaf error on dose distribution without changing MLC leaf positions in a TPS. The combination of EPID and the analysis software, such as the MLCQA plug-in option, can be additionally performed as a daily MLC positioning QA in the clinical machine QA program that provides the opportunity to detect the MLC positioning error effect on the VMAT plans before pretreatment patient-specific QA regardless of the treatment planning system. Our findings are congruent with the published data by Christophides et al., who investigated an automatic MLC QA method to define MLC errors ≥0.5 mm, stating that the detecting of an MLC error using an automatic MLC QA method presented in this study or their homemade software can reduce physicists’ and LINAC work-hours for patient-specific QA [30]. Furthermore, the numerical result of MLCQA with the gamma passing rate was used as an indicator and threshold to define the sensitivity of the patient-specific QA procedure for MLC position errors. In this study, the leaf-by-leaf analysis performed by MLC QA software provides quantitative data for the accuracy of each leaf pair; therefore, the passing rate of MLC positioning accuracy obtained by the evaluation of the picket fence test for error-free, Bank In, Bank Out, and Bank Both were 97%, 92%, 91%, and 87%, respectively. The results of MLCQA in the presence of the Bank In and Bank Out errors were relatively small dispositions to trigger LINAC from Beam On to Beam Off and were assumed as an acceptable value. The consequence of these MLC errors on SBRT/SRS even conventionally fractionated VMAT plans may not be noticed in the case of 3 mm/3% gamma criterion selection that is widely used for the evaluation of patient-specific QA. The current work indicates that systematic MLC leaf pair errors, such as a 1 mm MLC leaf positioning error causing the enlargement of the irradiated field at the picket fence test or a 1 mm MLC leaf positioning error causing narrowing of the irradiated field at the picket fence test, have a smaller but measurable impact on VMAT plans for SRS/SBRT than VMAT plans for the conventionally fractionated relatively complex plan such as head and neck and endometrium cases.

We suggest, based on our findings, that the passing rate of 95%-90% for the evaluation of the picket fence test by MLC QA could be used as a threshold for conventionally fractionated VMAT plans when the gamma index was selected as 2%/2 mm for the evaluation of patient-specific QA. As shown in Table 3, while the results of conventionally fractionated and high modulated VMAT plans’ QA, except a lung case evaluated with the 2%/2 mm criterion, are sensitive to reflect MLC errors, none of the SRS plans’ QA is not sensitive enough to reflect the MLC positioning error and only two of the SBRT plans’ QA resulted in a 68.3% and 69.3% and 68.8% and 69.5% passing rate in the presence of the Bank In and Bank Out errors, respectively. Even though more stringent analysis of the fluence with MLC positional error, such as 2% dose difference and 1 mm distance to an agreement to identify the accurate positioning of MLC, were suggested in the literature [10]. Our findings are consistent with previously published data that the gamma index of 2%/2 mm for highly modulated, multiple arc VMAT plans and arcs including large field sizes and more segment numbers, such as head and neck and endometrium cases as in this study, is sensitive enough to reflect MLC positioning errors even if they are as small or unable to trigger the machine from Beam On to Beam Off [21,26]. Although the criterion of 3%/3 mm has been typically used for gamma evaluation for patient-specific QA of both IMRT and VMAT plans, we concluded that the gamma index of 2%/2 mm, even 2%/1 mm rather than 3%/3 mm for patient-specific QA of SRS/SBRT, would be more clinically relevant criteria to identify the dose difference caused by MLC positioning errors.

The limitation of the study is that we could not conduct the proposed method for a long time to assess the characterization of MLC positioning errors and their impact on patient-specific QA. There is a strong positive linear relationship between MLC positioning and dose error in DVH of both IMRT and VMAT plans, for which we aim to conduct future work to analyze MLC positioning errors detected by a daily MLC QA picket fence test evaluation for a long-term period and its impact on the DVH of different clinical cases. Another limitation of this study was a selection of intentionally created 1 mm MLC positioning errors, even though the MLC QA software is very sensitive to detect MLC errors up to ±0.3 mm. Further investigation could be established to define tolerance for intentionally created <1 mm MLC positioning errors and analyze combining EPID with commercial MLC QA software that supports a tolerance level of ±0.3 mm for detecting MLC errors, giving their simplicity, efficiency, and accuracy. Therefore, it can be ideally used routinely for MLC QA as a morning machine QA.

## Conclusions

The present study shows that the detection of MLC positioning errors by analyzing picket fence tests acquired from EPID using an appropriate homemade or commercial software tool, such as MLC QA, is a fast and accurate way, and the implementation of this method into a morning machine QA procedure enables the reduction of time consumed for patient QA of IMRT/VMAT delivery when there is no time to employ patient-specific QA.
